# Identifying gaps in the continuum of care for cardiovascular disease and diabetes in two communities in South Africa: Baseline findings from the HealthRise project

**DOI:** 10.1371/journal.pone.0192603

**Published:** 2018-03-14

**Authors:** Alexandra Wollum, Rose Gabert, Claire R. McNellan, Jessica M. Daly, Priscilla Reddy, Paurvi Bhatt, Miranda Bryant, Danny V. Colombara, Pamela Naidoo, Belinda Ngongo, Anam Nyembezi, Zaino Petersen, Bryan Phillips, Shelley Wilson, Emmanuela Gakidou, Herbert C. Duber

**Affiliations:** 1 Institute for Health Metrics and Evaluation, University of Washington, Seattle, Washington, United States of America; 2 Medtronic Foundation, Minneapolis, Minnesota, United States of America; 3 Human Sciences Reseach Council, Cape Town, South Africa; 4 Public Health Institute, Global Health Fellows Program, Washington, DC, United States of America; The Chinese University of Hong Kong, HONG KONG

## Abstract

**Background:**

The HealthRise initiative seeks to implement and evaluate innovative community-based strategies for diabetes, hypertension and hypercholesterolemia along the entire continuum of care (CoC)-from awareness and diagnosis, through treatment and control. In this study, we present baseline findings from HealthRise South Africa, identifying gaps in the CoC, as well as key barriers to care for non-communicable diseases (NCDs).

**Methods:**

This mixed-methods needs assessment utilized national household data, health facility surveys, focus group discussions, and key informant interviews in Umgungundlovu and Pixley ka Seme districts. Risk factor and disease prevalence were estimated from the South Africa National Health and Nutrition Examination Survey. Health facility surveys were conducted at 86 facilities, focusing on essential intervention, medications and standard treatment guidelines. Quantitative results are presented descriptively, and qualitative data was analyzed using a framework approach.

**Results:**

46.8% of the population in Umgungundlovu and 51.0% in Pixley ka Seme were hypertensive. Diabetes was present in 11.0% and 9.7% of the population in Umgungundlovu and Pixley ka Seme. Hypercholesterolemia was more common in Pixley ka Seme (17.3% vs. 11.1%). Women and those of Indian descent were more likely to have diabetes. More than half of the population was found to be overweight, and binge drinking, inactivity and smoking were all common. More than half of patients with hypertension were unaware of their disease status (51.6% in Pixley ka Seme and 51.3% in Umgungundlovu), while the largest gap in the diabetes CoC occurred between initiation of treatment and achieving disease control. Demand-side barriers included lack of transportation, concerns about confidentiality, perceived discrimination and long wait times. Supply-side barriers included limited availability of testing equipment, inadequate staffing, and pharmaceutical stock outs.

**Conclusion:**

In this baseline assessment of two South African health districts we found high rates of undiagnosed hypercholesterolemia and hypertension, and poor control of hypercholesterolemia, hypertension, and diabetes. The HealthRise Initiative will need to address key supply- and demand-side barriers in an effort to improve important NCD outcomes.

## Introduction

Following public health successes in the prevention and treatment of communicable diseases, and combined with increasing life expectancy in most countries, non-communicable diseases (NCDs) have become the leading cause of death globally, accounting for nearly 40 million deaths in 2015.[1] The burden of NCDs has disproportionately increased in low- and middle-income countries (LMICs), which are also more likely to face challenges due to developing health systems and limited infrastructure.

To advance community-based solutions to improve NCD outcomes in underserved populations, a broad coalition of partners from communities, governments and donors launched HealthRise, a five-year demonstration project designed to increase detection, management, and control of diabetes and cardiovascular diseases (CVD) in support of—and together with—local health systems and communities in four countries: South Africa, India, Brazil, and the United States. The initiative builds on World Health Organization (WHO) and Sustainable Development Goals (SDGs) objectives, which emphasize NCDs as a global priority and recommends the use of effective community-based interventions.[2,3] HealthRise seeks to further this mission by implementing and evaluating innovative community-based strategies for diagnosing, treating, and managing CVD and diabetes, thereby, creating an evidence base for strategies that improve health outcomes. [4]

In South Africa, one of the HealthRIse supported countries, recent policy reforms have begun to address the increasing burden of NCDs, but there remain significant opportunities to expand access to NCD care and treatment, and increase the availability of effective community-based services and pharmacological interventions. [5–7] Consistent with the strategies outlined by the WHO Global NCD Monitoring Framework, South Africa released its Strategic Plan for the Prevention and Control of Non-Communicable Disease 2013–2017.[2,8] However, only three South African provinces have drafted their own NCD strategic plans, and none have been approved thus far.[9] Buy-in at the provincial and local levels will be critical for South Africa to address chronic disease management by capitalizing on its successes with community-based interventions focused on diagnosis and long-term management of HIV.

In addition to obtaining provincial and local buy-in of NCD plans, numerous other obstacles need to be overcome and/or addressed. Among the documented challenges South Africa’s health system faces are human resource shortages, insufficient health care financing, and inadequate public health system infrastructure.[10–12] Addressing these gaps requires greater evidence and prioritization of solutions that address the current and future burden of disease.[13] Furthermore, social disparities have posed a persistent challenge to past community-based interventions. However, these disparities can be combatted and mitigated with interventions informed by the present needs of specific communities.[14,15] Among the strategies suggested is the creation of a more formal cadre of community health workers (CHWs) within district-based primary care teams, a key focus of the HealthRise initiative. [16,17]

The HealthRise initiative presents an opportunity to create evidence-driven community-based initiatives at a critical time. The first step in developing this evidence base is establishing baseline community findings. In this paper, we present results from the HealthRise needs assessment conducted in two communities in South Africa, Umgungundlovu and Pixley ka Seme. We identify baseline characteristics and barriers to care for hypertension, hypercholesterolemia, and diabetes in order to inform HealthRise community-based interventions to control these conditions. Our analysis examines the burden of NCDs and contributing risk factors, and highlights gaps in diagnosis, treatment, and management of these conditions from both the patient and provider perspective. Finally, we briefly present the demonstration projects that will be implemented in each district to address the needs assessment findings.

## Methods

**Fig 1:**
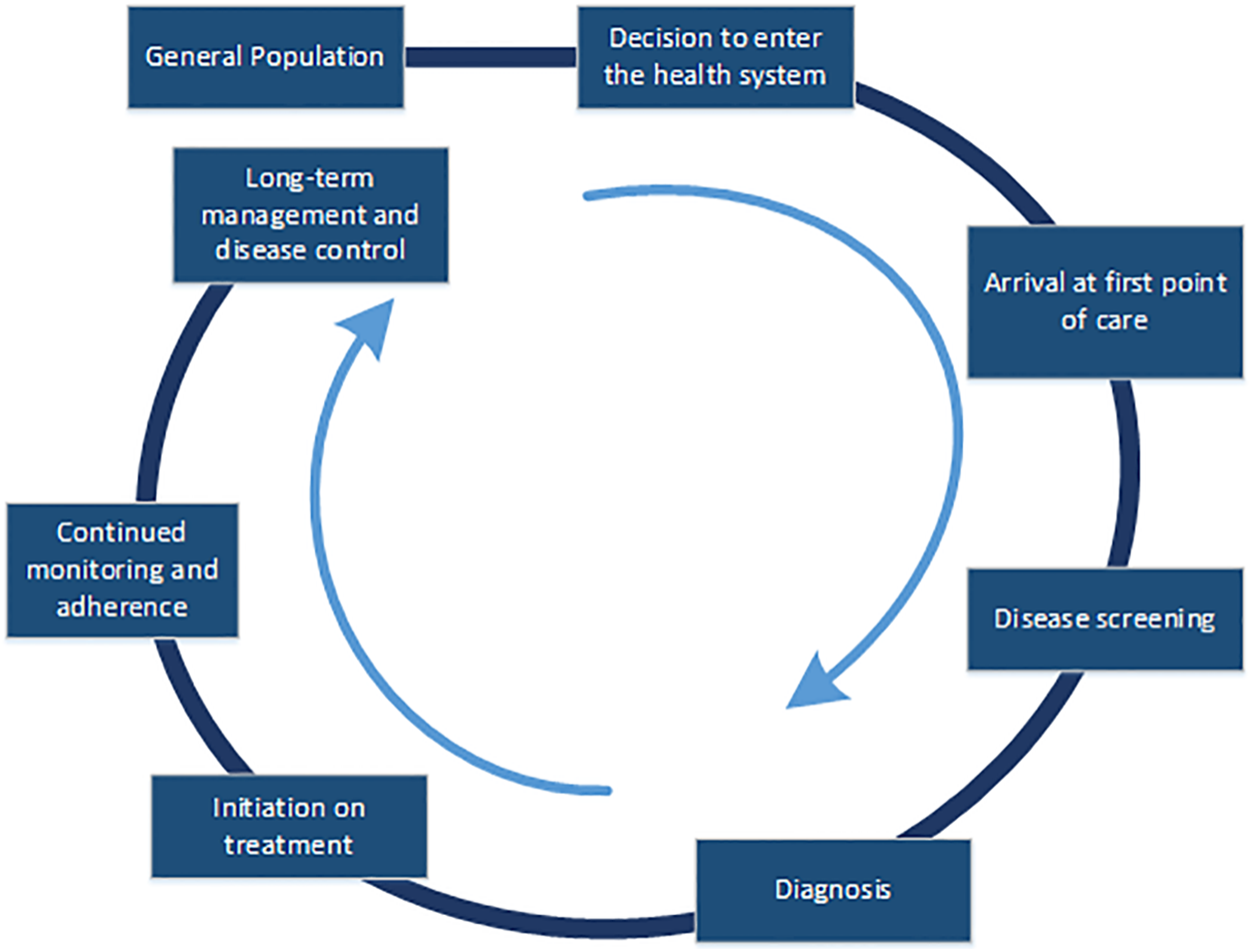
The continuum of care framework.

To understand the burden and current response to NCDs in South Africa, we examined barriers to diagnosis, treatment, and management of NCDs. We used a continuum of care (CoC) framework to understand how an individual moves through and interacts with the health care system (Fig 1). [4]

### Study setting and population

The HealthRise project is taking place in two districts within South Africa—Umgungundlovu in KwaZulu-Natal Province, and Pixley ka Seme in the Northern Cape Province. These districts were chosen by key stakeholders given their involvement in the piloting of the National Health Insurance scheme, their status as underserved districts, and the presence of willing and interested government and nongovernmental partners. Both districts demonstrate the complex heterogeneity of South African society, with its enormous inequalities in wealth, often along racial lines, that along with other social determinants result in significant health disparities. Both districts also reflect the rapid social and epidemiologic transition underway in South Africa, and provide a microcosm of the ethnic diversity with the country’s population.[1,18]

### Data

This mixed-methods needs assessment utilized previously collected national household data, as well as primary data collected through health facility surveys, qualitative focus group discussions (FGDs), and key informant interviews (KIIs) in each of the two districts of interest.

To quantify risk factor and disease prevalence, and explore health-seeking behavior, we used the South Africa National Health and Nutrition Examination Survey (SANHANES) completed in 2011 and 2012. This survey included a questionnaire, physical examination, and biomarker measurement. Because SANHANES was representative at the provincial level, we used district-level population structures and demographic information from the 2011 Census to scale SANHANES estimates to the district level.

Public health facility sample frames were obtained from the provincial Ministry of Health offices of KwaZulu-Natal and Northern Cape. Comprehensive lists of private providers in the districts were not available. Instead, a convenience sample frame was constructed using online searches for private providers located within the districts. In Pixley ka Seme, a sparsely populated area, all health facilities within the district were included. In Umgungundlovu, where there were more facilities, we sampled facilities from four randomly selected sub-districts, stratifying by facility type. Verbal consent from the facility administrator was required for participation. In Umgungundlovu, 15% of selected samples were replaced due to refusals and administrative barriers (e.g. unavailable supervisors to grant permission). These sample replacements targeted facilities of the same type, where possible, and were conducted through random selection.

The distribution of platform type for the facility sample is presented in Table 1. Surveys were conducted at 86 facilities and based on the WHO package of essential NCD interventions as well as South Africa’s Essential Medicine List and Standard Treatment Guidelines for Primary Health Care 2014.[19] The survey was adapted from a questionnaire developed for the Access, Bottlenecks, Costs, and Equity study conducted by the Institute for Health Metrics and Evaluation (IHME).[20] Surveys were translated into English, Afrikaans, and Zulu. Information was collected about facility capacity, equipment availability, pharmaceutical and supply stocks, staffing, and services provided. Data was collected on netbooks via computer-assisted personal interviews (CAPI) using SurveyBe Implementer version 4.3.31 software, and de-identified data was stored for analysis.

**Table 1:** Facility sample by district and platform.

Platform	Umgungundlovu	Pixley ka Seme
Hospital	3	3
Community Health Centre (CHC)	6	8
Primary Health Centre (PHC)	18	28
Mobile clinic	9	0
Satellite clinic	0	4
General private practitioner clinic	3	0
Private pharmacy	4	0
Total	43	43

Qualitative data were collected through semi-structured KIIs and FGDs with target populations, stakeholders, and health system personnel. Populations of interest include those identified by the continuum of care. Key stakeholders, and ultimately key informants, were identified through discussions with the District Health Office leadership and officials at participating health care facilities. Following receipt of verbal consent in the native language of each respondent, individual interviews were conducted with 21 healthcare providers and policymakers, including doctors, nurses, CHWs, and health officials, as described in Table 2. Additionally, 15 FGDs were conducted with NCD patients as well as members of the general community population. Patients were recruited from different facilities, including attendees of both public and private facilities within the same group (where possible), in order to represent a range of socio-demographic backgrounds. Focus group discussions were facilitated by contracted, trained interviewers with qualitative survey expertise, who were fluent in English, isiZulu, and isiXhosa in Umgungundlovu; and English, Afrikaans, isiXhosa, and Setswana in Pixley ka Seme. The transcripts were translated into English in preparation for qualitative analysis.

**Table 2:** Interview and focus group participants.

Interviewees	Umgungundlovu	Pixley ka Seme
CCGs* or CCG managers	2 interviews	1 interviews
Healthcare Providers: PHC doctors, Public GP**, Private GP	6 interviews	3 interviews
Health facility managers or policy maker	1 interviews	2 interviews
NGO Officials	1 interview	1 interview
Nurses	2 interview	3 interviews
General Community Members	5 FGD	4 FGD
NCD Patients	4 FGD	2 FGD
Total	11 interviews / 9 FGD	10 interviews / 6 FGD

*CCG-community care giver

**GP-general practitioner

### Analysis

SANHANES data were used to estimate prevalence of disease and risk factors as well as diagnosis, treatment, and control of hypertension, hypercholesterolemia, and diabetes mellitus.[21] SANHANES data, however, cannot be used directly to make estimations at the district level as it was not sampled to be representative at this level. We instead made estimates of stratified subpopulations (sex, race, and sex-race combinations) at the national level and then weighted the estimates to match the district level population structure from the 2011 Census.[22,23] We were unable to stratify by age due to small sample sizes, but the national and district means and confidence intervals were all within one year of each other suggesting comparability. We present district level continuum of care estimates. However, due to small sample sizes and increased uncertainty, we do not present district-level estimates of subpopulations. Instead, subpopulation estimates are reported at the national level, adding context and value to district level disease prevalence estimates.

Individuals were classified as having disease if a patient reported a history of prior diagnosis or were found to have a biomarker measurement above recommended thresholds. Individuals were considered to be hypertensive if they had a diastolic blood pressure ≥ 90 mmHg or systolic blood pressure ≥ 140 mmHg.[24] For diabetes, the threshold was HbA1c level ≥ 6.5%.[25] For high cholesterol, the threshold was non-fasting total serum cholesterol levels ≥ 6.2 mmol/l.[26] The same threshold was used for determination of both diagnosis and control for hypertension. For diabetes, control was defined as having a HbA1c < 8%. As there is no agreed upon treatment goal for cholesterol, we did not estimate disease control for hypercholesterolemia.[27] Individuals were considered under treatment if they reported receiving allopathic treatment for the condition. In reporting their condition, patients were not asked to differentiate types of cholesterol; thus, we considered those that reported receiving anti-lipid medications as under treatment, though we recognize that anti-lipid medications are not prescribed for all individuals with high cholesterol. We made estimates of prevalence for four risk factors: binge drinking (consuming ≥ 5 drinks for men or ≥ 4 drinks for women in one sitting); body mass index (BMI) (overweight: ≥ 25 kg/m^2^ and obese: ≥ 30 kg/m^2^); smoking (currently using any type of tobacco); and physical inactivity (<600 metabolic equivalents (METS) per week). We assessed for differences between groups using a survey weighted chi-square test.

Facility data were analyzed separately by district and platform type. To analyze availability of pharmaceuticals and equipment, physician input and national guidelines were utilized to group items based on condition.[19,28] Table 3 contains a list of testing equipment, pharmaceuticals, and guidelines included for each condition. To calculate availability, we summed the number available in each category (pharmaceutical, equipment, guidelines) at the facility and divided by the total number of items listed.

**Table 3:** Items included in facility capacity analysis.

Category	Diabetes	Hypertension
Pharmaceuticals	insulin, aspirin, statins, ACE Inhibitors, biguanides and sulphonylureas	insulin, aspirin, statins, ACE Inhibitors, thiazide diuretic, calcium channel blockers and beta blockers
Testing Capacity equipment	scale, blood pressure apparatus, plasma glucose tests or glucometers and blood glucose strips, urinalysis, total cholesterol test or fasting lipid profile or LDL cholesterol test, creatinine test (eGFR) and HbA1c test	scale, blood pressure apparatus, plasma glucose tests or glucometers and blood glucose strips, creatinine tests (eGFR), urinalysis and total cholesterol test or fasting lipid profile or LDL cholesterol test.
Guidelines	PC101 or disease specific treatment guidelines and EML.	PC101 or disease specific treatment guidelines and EML.

The framework approach was used to analyze qualitative data.[29] Initially, a representative sample of KIIs and FGDs were reviewed. During this process, open coding was used to classify participant responses. After reading and coding the initial group of interviews, a working thematic framework was developed based on emerging themes. The remaining interviews were coded based on the working thematic framework.

### Ethics review

This study was approved by the Institutional Review Board of the University of Washington and the Ethical Review Board of the University of Witwatersrand, as well as the provincial research boards of KwaZulu-Natal and Northern Cape.

## Results

### Population characteristics, disease prevalence and risk factors

Race, age and gender characteristics can be found in Table 4. As mentioned above, Umgungundlovu is predominantly Black African (84.8%), while Pixley ka Seme is majority Coloured (59.4%). Both districts have similar age distributions, with approximately two-thirds of the population between ages 15 and 64. Just over half of the population was female in Umgungundlovu and Pixley ka Seme.

**Table 4:** Population characteristics and risk factors.

	Umgungundlovu % (95% CI)	Pixley ka Seme % (95% CI)
Race Group		
Black African	84.8 (84.5,85.0)	30.8 (30.1,31.5)
Coloured	2.1 (2.0,2.2)	59.4 (58.7,60.2)
Indian or Asian	6.7 (6.5,6.9)	0.5 (0.4,0.6)
White	6.2 (6.1,6.4)	8.4 (7.9,8.8)
Age Distribution		
0–14	28.2 (27.9,28.5)	31.7 (31.0,32.4)
15–64	66.5 (66.1,66.8)	62.2 (61.5,63.0)
65+	5.4 (5.2,5.5)	6.0 (5.7,6.4)
Female	52.3 (52.0,52.6)	50.9 (50.1,51.7)
Disease Prevalence		
Diabetes	11.0 (7.2,14.9)	9.7 (5.1,14.2)
Hypertension	46.8 (41.2,52.3)	51.0 (44.3,57.6)
Hypercholesterolemia	11.1 (7.4,14.8)	17.3 (10.9,23.7)
Risk Factors		
Binge drank alcohol in past week	19.9 (15.3,24.5)	26.7 (20.2,33.2)
Inactive	40.5 (33.7,47.3)	39.1 (31.1,47.1)
BMI ≥ 25	55.3 (49.8,60.8)	54.8 (47.3,62.4)
Current smoker	18.1 (13.5,22.8)	35.2 (27.8,42.6)

* Derived from 2011 municipal census data (23, 24).

^ Estimated using 2012 South Africa National Health and Nutrition Examination Survey (SANHANES)

Hypertension was the most prevalent condition among the three examined in this study (Table 4). We estimated that approximately half of the population had elevated blood pressure (46.8% in Umgungundlovu and 51.0% in Pixley ka Seme). Both diabetes and high cholesterol were much less common, with prevalence of diabetes near 10% in both districts, and hypercholesterolemia estimated at 11.1% and 17.3% in Umgungundlovu and Pixley ka Seme, respectively. Nationally, women were found to have a statistically significant higher estimated prevalence of diabetes compared to men (12.0% vs 7.5%, p = 0.002). No statistically significant gender difference was noted for hypercholesterolemia or hypertension. The Indian population had the highest prevalence of diabetes nationally, with prevalence estimated at 22.6%. For other diseases, the differences between groups were less stark, although black Africans had a significantly lower prevalence of high cholesterol than other populations (24.1% vs. 7.9%, p<0.005).

Elevated body-mass index (BMI) was the most prevalent risk factor (Table 4). An estimated 55.3% and 54.8% of individuals were overweight or obese in Umgungundlovu and Pixley ka Seme, respectively. Nationally, 77.9% of overweight persons did not try to lose weight, and 61.5% of overweight persons reported being happy with their current weight. The prevalence of obesity among women was more than twice that of men. Despite this high prevalence, less than half of overweight women or men described themselves as overweight. Prevalence of self-reported physical inactivity was approximately 40% in each district, with women more inactive than men (44.1% vs 36.0%) nationally. Individuals with high cholesterol or diabetes were more likely to be overweight than the general population. Smoking and binge drinking, although less prevalent than overweight and obesity, were highly prevalent among men (33.8% and 33.2% respectively). In qualitative interviews, alcoholism was a major concern among community members and health care providers.

### Continuum of care

**Fig 2:**
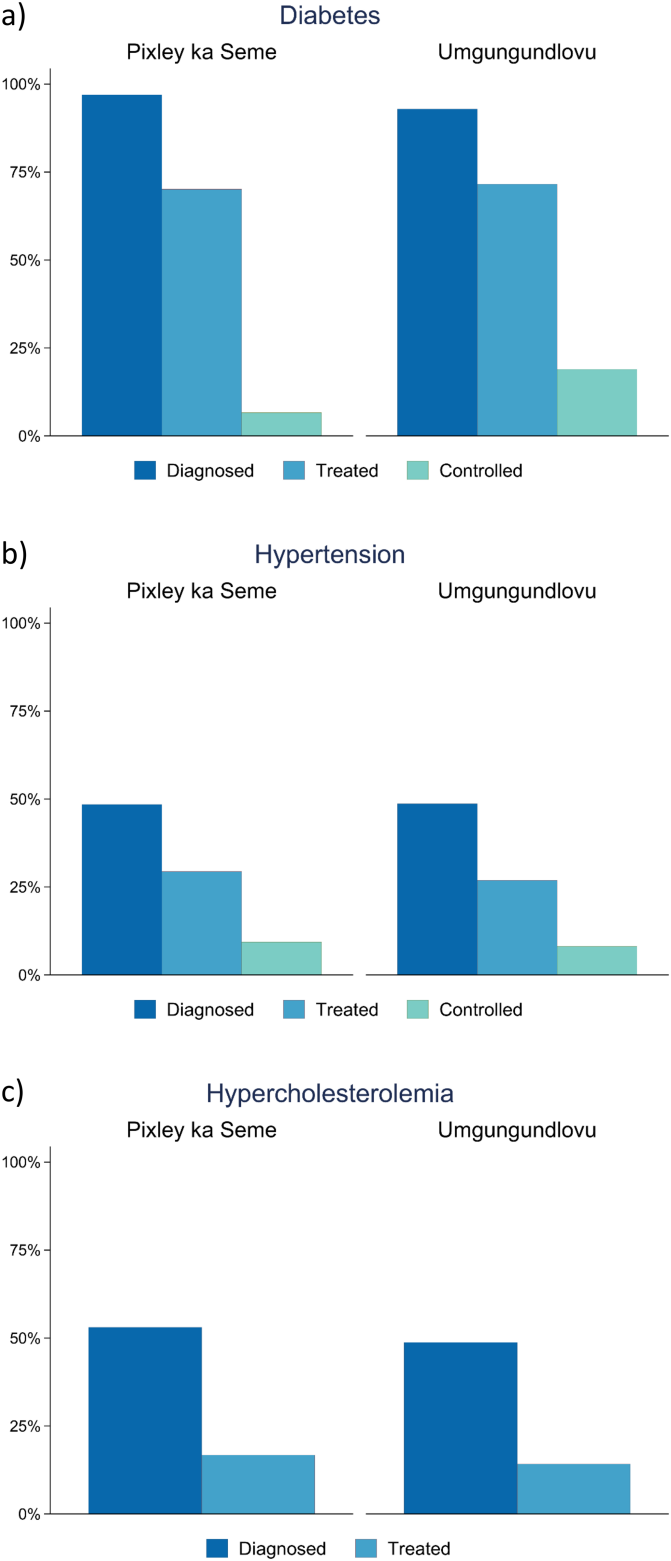
Estimating the continuum of care for (a) diabetes, (b) hypertension and (c) hypercholesterolemia in Umgungundlovu and Pixley ka Seme.

In both districts, more than 45% of individuals with hypertension and high cholesterol were undiagnosed (Fig 2). For those with positive diabetes biomarkers, 10% were undiagnosed in Pixley ka Seme and 25% were undiagnosed in Umgungundlovu.

At the national level, pronounced differences were noted among several subpopulations. Comparing urban and rural areas, 56.0% of those with high cholesterol received a diagnosis in urban areas, versus a mere 31.8% in rural areas. We also note variation in diagnostic rates by race. Looking at hypercholesterolemia, we found that 54.9% of Whites had been diagnosed, compared to 52.8% of Coloureds, 48.2% of black Africans, and 38.1% of Indians/Asians. Compared to other races, Indians/Asians with diabetes also had the lowest percentage (63.6%) of having received a prior diagnosis. Interestingly, we found the opposite pattern when examining hypertension. The percentage of Indians/Asians with hypertension who had received a prior diagnosis was highest (57.9%) and the percentage of Whites was lowest (42.7%). Women and men were found to have similar rates of prior diabetes diagnosis (75.4% vs 80.1%, p = 0.542). However, a larger percentage of women had received a diagnosis of hypertension (55.7%) compared to men (40.5%, p<0.001).

Among individuals diagnosed with either hypertension or diabetes, most received some form of allopathic treatment (Fig 2). At the same time, only a fraction of these individuals were found to have condition-specific biomarkers considered to be within recommended levels of control at the time of the survey. Among those on treatment for hypertension, 31.7% in Pixley ka Sems and 30.2% in Umgungundlovu were found to have blood pressures less than 140/90mmHg. A smaller percentage of individuals with diabetes were found to have controlled disease-9.4% in Pixley ka Seme and 26.4% in Umgungundlovu. In contrast, those with high cholesterol were less likely to be treated, but were more likely to control their disease once treatment was initiated. Only 16.7% of individuals with hypercholesterolemia in Pixley, and 14.2% in Umgungundlovu, reported being on treatment.

### Outreach and care-seeking behaviors

Qualitatively, many patients reported being unaware of the signs and symptoms of NCDs, and few reported knowing that these conditions are often asymptomatic in the early stages. Additionally, CHWs reported a lack of training and equipment needed to screen or test for disease or monitor control in individuals’ homes, necessitating patient visits to a facility. Providers expressed concern that CHWs lacked integration with facility staff and processes, and felt that outreach efforts for NCDs were underfunded and understaffed. Interviews suggested that patients frequently waited to seek care or diagnostic services until severe symptoms presented. Reasons for delaying care-seeking behavior included fear of being diagnosed with HIV, lack of transportation to the facility, concerns about lack of confidentiality, perceived discrimination and preferential treatment for certain racial groups at some facilities, and distrust of the motives of healthcare staff, particularly nurses. When patients did seek care at facilities, they reported frustration with high patient volumes and long wait times at public facilities. Once patients had been diagnosed, barriers to treatment and control of disease stemmed from patients’ lack of knowledge about how to monitor their disease, and lack of access to blood pressure and blood glucose monitoring equipment. Patients also reported being unable to make diet modifications due to financial constraints.

### Supply-side barriers

**Fig 3:**
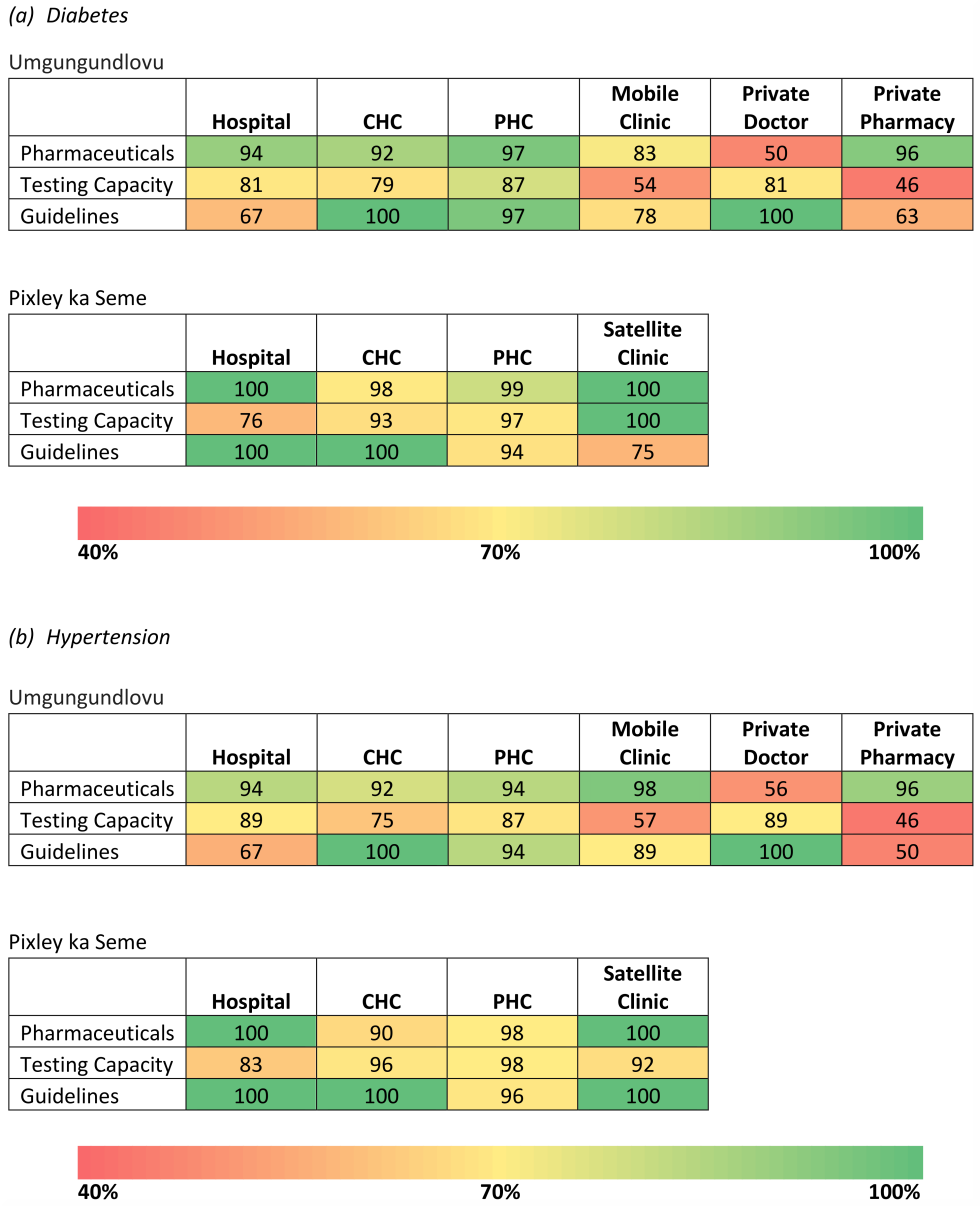
Percent of items available at facilities for (a) diabetes and (b) hypertension as per national guidelines.

In Umgungundlovu, there was limited availability of testing equipment (Fig 3). Around 20% of hospitals and community health centers (CHCs) lacked blood glucose test strips or plasma glucose tests on the day of the survey. Over 40% of PHCs lacked total cholesterol tests and 56% of PHCs lacked LDL cholesterol tests. In order to fully diagnose patients, providers reported needing to refer patients to receive more accurate tests from other facilities, thereby increasing the time between first contact with the patient and diagnosis. Additionally, providers suggested equipment was in substandard condition and that high patient volumes interfered with their ability to coach patients on strategies for managing their condition. Although our facility survey did not find stock-outs of the required classes of pharmaceuticals for CVD and diabetes within the public sector, there was widespread belief among community members and providers that stock-outs were common.

Providers confirmed that long wait times identified in patient interviews were indeed a deterrent to care-seeking behavior and resulted in reduced patient interest in returning to facilities for condition monitoring. Despite the emphasis patients and providers placed on long wait times, there existed only moderate patient volumes at the time of the survey.

## Discussion

This study aimed to quantify the continuum of care—prevalence, diagnosis, treatment, and control—for diabetes, hypertension, and hypercholesterolemia in two South African districts, Pixley ka Seme and Umgungundlovu. Simultaneously, we sought to identify key barriers within the continuum of care, thereby providing critical information on needs and targets for community-based interventions.

Looking at the initial steps in the continuum of care, we found markedly high rates of risk factors for NCDs in both districts, particularly overweight/obesity, consistent with findings from adult populations in other LMICs.[30,31] For hypertension and hypercholesterolemia, we found low levels of diagnosis, indicating that most patients were unaware of their disease. This finding is in stark contrast to the rates of diabetes diagnosis, which have been found to relatively high in other settings as well.[32] One possible explanation for this discrepancy is that patients are much more likely to be symptomatic from diabetes. Although literature on this is limited, a study from the United States, where diabetes screening is much more prevalent, found than one-third of patients with diabetes were symptomatic at the time of diagnosis.[33] Interviews suggest limited knowledge regarding the asymptomatic nature of hypertension and hypercholesterolemia, and HealthRise interventions will need to address this early gap in the continuum of care.

Our estimates of hypertension prevalence, around half of the adult population in both Umgungundlovu and Pixley ka Seme, are in line with those previously reported in South Africa. In a 2014 study, Lloyd-Sherlock et. al. estimated a prevalence of 78% among South Africans over the age of 50.[34] Another study from Limpopo Province, South Africa, found a prevalence of 41% among the adult population 15 and over, with a clear association with age.[35] It should also be noted that our estimates of hypertension prevalence were found to higher than in most other LMICs.[34,36–38] These findings are in contrast to our diabetes and hypercholesterolemia estimates, which were generally lower than other comparable countries.[37,38]

Low rates of control were notable among those with hypertension, hypercholesterolemia and diabetes, and comparable to prior estimates in South Africa.[39,40] The fact that such a small percentage of patients have well-controlled disease is not a surprise given that the study was performed in districts that are historically underserved. These findings further support the importance of the future HealthRise demonstration projects.

We also identified a number of barriers that can be addressed through multi-sectoral partnerships, such as the proposed HealthRise demonstration projects. From a facility perspective, limited human resource capacity and availability of diagnostic equipment at lower-level facilities were identified as critical barriers. In addition, patients who attended public facilities reported long waiting times and insufficient consultation time with providers. This may imply that disease burden has outpaced the capacity of the health system to provide efficient care. By extending the health care system to CHWs, and further integrating NCD care, we believe that many of these barriers can be overcome.

Importantly, the South African government and other partners have already begun to draw from lessons learned during the HIV epidemic. Specifically, much can be gained from prior experiences of scaling up HIV services and integrating and decentralizing HIV care and other service lines. Looking critically at what has worked, and determining its applicability to the diagnosis and treatment of NCDs, will be essential.[41–44] In July 2016, South Africa hosted the International AIDS Conference, which featured discussions on NCD integration at the community and primary care level by leveraging existing platforms for HIV and TB detection, care, and management.[45] Continued dialogue and research will help grow the evidence base for NCD care in South Africa.

To address the gaps identified in the NCD continuum of care, the HealthRise project is implementing community-based interventions and a prospective program evaluation in each district. The interventions support the Department of Health’s Integrated Clinical Services Management Model, through the training and empowering of CHWs. CHWs will provide screening, promote follow-up and linkages to care, and coordinate NCD support groups, educational classes, and other social services. The interventions will also look at developing innovate new ways to improve linkage and retention through programs such as savings and loan groups, and income and/or food generating group gardens. These programs aim to ensure that patients have the financial and social resources necessary to access treatment and manage their disease after diagnosis.

This baseline needs assessment is the first step in a rigorous evaluation process. The HealthRise approach provides a unique opportunity to prospectively evaluate the impact of a public health program. A follow-up endline evaluation will similarly assess the HealthRise interventions along the entire continuum of care. The generation of additional evidence on community-based interventions for the management of NCDs will allow policy makers to determine the utility of intervention scale-up.

### Limitations

This study should be viewed within its limitations. First, the SANHANES data were representative at the province level, requiring that we use Census information on the demographic composition of each district in order to scale results to the district level. Additionally, samples used to estimate diagnosis, treatment, and control by population group (e.g., race, population density, etc.) were relatively small and must be interpreted cautiously. The biometric portion of SANHANES had significantly lower response rates, and rates differed by race and socioeconomic status, making it difficult to compare disease status between these groups. Nevertheless, such low levels of diagnosis for hypertension and hypercholesterolemia should be considered indicative of a significant problem. Facility-level data was particularly challenging due to low response rates from private facilities in Umgungundlovu, weakening our power to draw conclusions between public and private facilities. Lastly, when gathering information about pharmaceuticals, we asked facilities to report whether any pharmaceutical in a class was available at the facility. We did not ask about specific pharmaceuticals, thus limiting our ability to comment on the availability and stock-out of specific medications.

## Conclusion

We identified important gaps in the diagnosis of hypercholesterolemia and hypertension, and the treatment of hypercholesterolemia, hypertension, and diabetes. Furthermore, we elucidated key barriers, both supply and demand, that can be addressed through the HealthRise community-based interventions. If South Africa is to meet the Sustainable Development Goal of reducing premature mortality due to NCDs by one third by 2030, considerable progress must be made. Working with provincial governments, local partners will utilize their vast knowledge and insight, much of it from the HIV epidemic, to develop ways to extend health services through the use of CHWs, integrate NCD care into other health services, and improve the capacity of health facilities to diagnosis and treat NCDs. Upon completion of the demonstration projects, the impact evaluation will provide important evidence regarding the potential for these interventions to be scaled up and serve as a model for future interventions, nationally and internationally.
